# Ultrasound common hepatic artery denervation (CHA-D) as a novel neuromodulatory strategy for cardiovascular-kidney-metabolic syndrome: first-in-human experience

**DOI:** 10.1007/s00392-026-02949-9

**Published:** 2026-06-03

**Authors:** Mert Tokcan, Franciska Diana Wolter, Mathias Hohl, Peter Fries, Thorsten Kessler, Saarraaken Kulenthiran, Michael Böhm

**Affiliations:** 1 Klinik für Innere Medizin III – Kardiologie, Angiologie und Internistische Intensivmedizin, Saarland University Medical Center, Saarland University, Homburg, Germany; 2 HOMICAREM (HOMburg Institute of CArdioREnalMetabolic Medicine), Medical Faculty, Saarland University, Homburg, Germany; 3 Klinik Für Diagnostische Und Interventionelle Radiologie, Saarland University Medical Center, Saarland University, Homburg, Germany

**Keywords:** Common hepatic artery denervation, Hypertension, Cardiovascular-kidney-metabolic syndrome, Sympathetic nervous system, Cardiometabolic

## Abstract

**Background:**

Hypertension may remain uncontrolled despite lifestyle measures, guideline-directed multidrug therapy, and renal denervation (RDN). Sympathetic overactivity contributes to persistent blood pressure (BP) elevation and cardiometabolic dysfunction. Given its central role in metabolic regulation and dense sympathetic innervation, the liver represents an attractive target for interventional neuromodulation.

**Methods:**

This first-in-human ultrasound common hepatic artery denervation (CHA-D) was performed in a patient with recurrent uncontrolled hypertension and cardiometabolic comorbidity despite prior RDN and limited medical treatment options. Follow-up included ambulatory and office BP monitoring at 1 and 6 months, proton density fat fraction magnetic resonance imaging, metabolic parameters, and patient-reported outcomes (SF-36, EQ-5D-5L). In addition, current clinical studies evaluating CHA-D and multi-organ denervation were reviewed.

**Results:**

The procedure was completed successfully without intraprocedural complications. No hepatobiliary, pancreatic, or renal safety signal emerged during follow-up. Mean 24-h BP decreased from 179/88 mmHg at baseline to 151/87 mmHg at 1 month and 139/83 mmHg at 6 months, corresponding to systolic BP reductions of 28 and 40 mmHg, respectively, without intensification of antihypertensive therapy. Office BP decreased from 176/116 mmHg at baseline to 125/85 mmHg at 6 months. Hepatic fat fraction declined from 16.2% to 11.5%, HbA1c from 6.2% to 5.9%, insulin requirement was reduced, and body weight decreased by 8 kg. Patient-reported outcomes also improved during follow-up.

**Conclusion:**

This first-in-human experience suggests CHA-D as a technically feasible and safe neuromodulatory approach with convergent signals of hemodynamic and cardiometabolic benefit, supporting further prospective evaluation of CHA-D in cardiovascular-kidney-metabolic syndrome.

**Graphical Abstract:**

**Figure:**
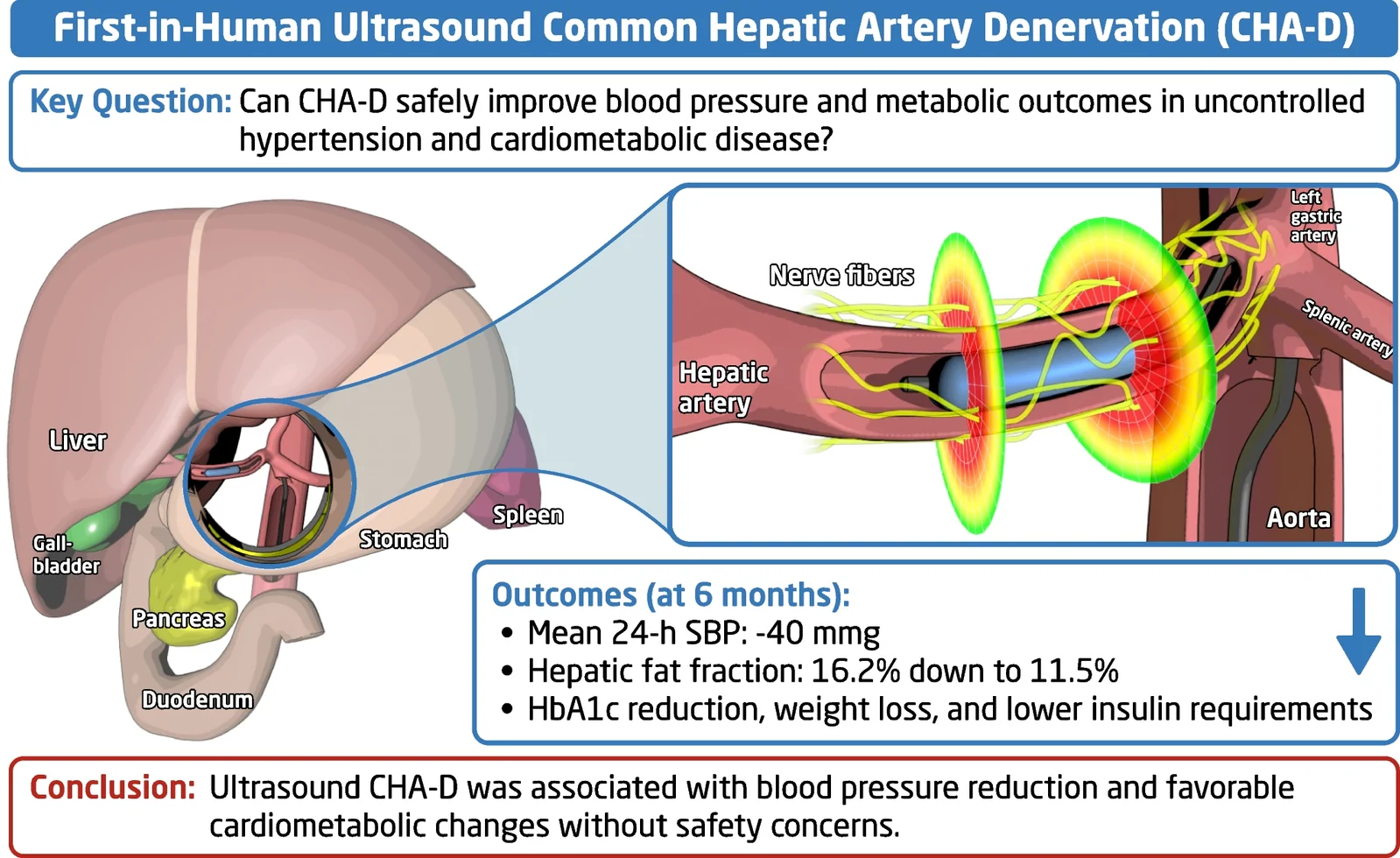

## Introduction

Hypertension is the most prevalent and potent contributor to cardiovascular morbidity and mortality [1]. However, a considerable proportion of patients fail to achieve guideline-recommended blood pressure (BP) targets despite lifestyle modification and multidrug antihypertensive therapy [2, 3]. Uncontrolled hypertension is characterized by heightened sympathetic nervous system activity [4], which contributes to persistent BP elevation and metabolic dysregulation promoting target-organ damage [5, 6]. Catheter-based renal denervation (RDN) is a guideline-approved device-based option for selected patients with uncontrolled or resistant hypertension, supported by sham-controlled trials demonstrating consistent BP reductions [2, 7–11]. Beyond BP reduction, translational evidence suggests that RDN may also exert favorable effects on cardiometabolic parameters including fasting glucose and hepatic steatosis [12, 13]. The liver represents an attractive target as it is the central organ of glucose and lipid homeostasis and is richly innervated by sympathetic fibers [14]. In experimental models, interruption of hepatic sympathetic signaling has been associated with reduced hepatic steatosis and improved glucose tolerance [15, 16]. Preclinical studies of combined RDN and common hepatic artery denervation (CHA-D) further support this concept [17, 18], and early clinical observations suggest that CHA-D may provide additional BP lowering and enhance the effects of RDN [19, 20].

This study presents the first-in-human use of ultrasound CHA-D in a non-responder to RDN with uncontrolled hypertension and a high cardiometabolic risk profile. The procedure, periprocedural safety, and longitudinal follow-up are described, including BP, laboratory parameters, and proton density fat fraction magnetic resonance imaging (PDFF-MRI). The case is further placed in the context of ongoing clinical studies evaluating CHA-D and multi-organ denervation strategies.

## Methods

### Patient selection

Ultrasound CHA-D was performed as an individual therapeutic attempt in a patient with symptomatic uncontrolled arterial hypertension and limited pharmacological treatment options owing to multiple intolerances and allergies to antihypertensive medications. Early target-organ damage and pronounced cardiometabolic comorbidity further underscored the severity of the clinical condition. The procedure was undertaken after established treatment strategies, including lifestyle measures, pharmacotherapy, and previous RDN, had failed to provide permanent BP control. The indication for CHA-D was determined by an interdisciplinary team with expertise in hypertension management and endovascular interventions. Potentially reversible contributors and secondary causes of hypertension were assessed and excluded as clinically appropriate. After detailed discussion of the experimental nature of the procedure and its potential risks, the patient provided written informed consent for the intervention and for publication. According to local regulations, formal ethics committee approval was not required for this compassionate-use single-case intervention. All procedures performed were in accordance with local ethical standards and with the Declaration of Helsinki and its later amendments.

### Pre-procedural workup

**Fig. 1:**
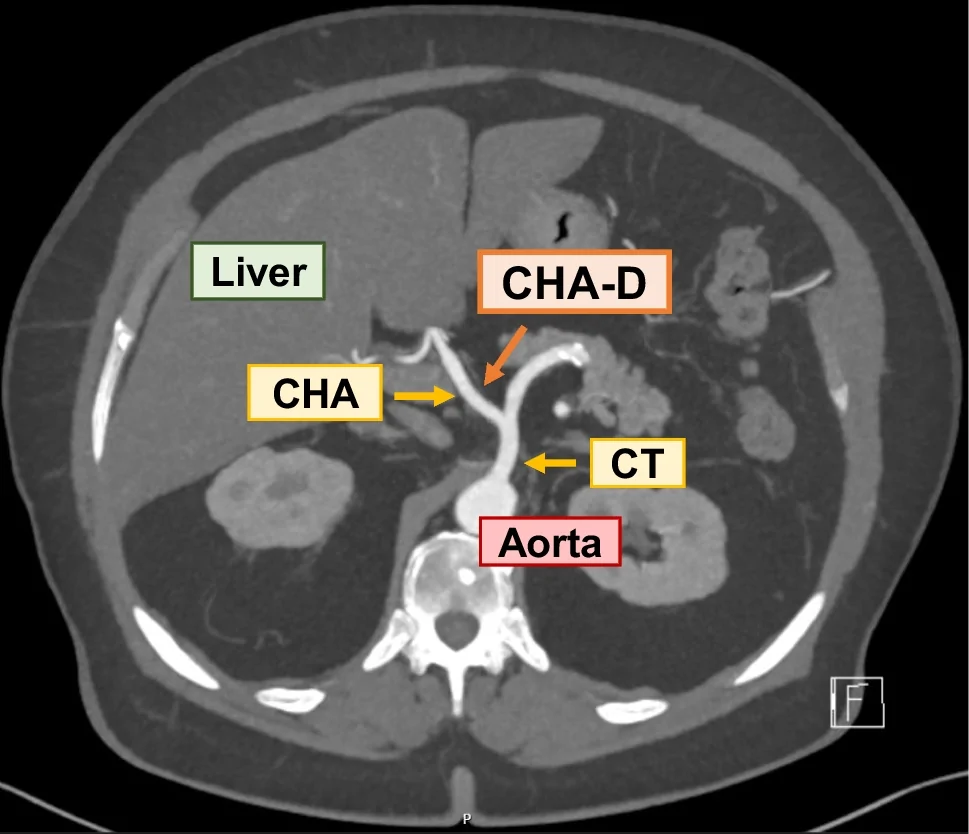
Pre-procedural imaging of the common hepatic artery. Contrast-enhanced computed tomography angiography demonstrating the anatomy of the celiac trunk and common hepatic artery. The imaging was used to assess anatomical eligibility, exclude relevant stenoses or variants, and guide procedural planning for hepatic artery denervation. CHA, common hepatic artery; CHA-D, common hepatic artery denervation; CT, celiac trunk

Anatomical eligibility was assessed by contrast-enhanced computed tomography angiography to identify celiac–hepatic variants and to exclude relevant stenoses, dissections, aneurysms, or other findings that could compromise safe catheter navigation and treatment planning (Fig. 1). Because preclinical data suggest potential effects of CHA-D on fatty liver disease [15], liver steatosis was quantified using PDFF-MRI. Health status and quality of life were evaluated using the EQ-5D-5L and SF-36 questionnaires [21, 22]. Secondary causes of hypertension had been excluded previously, and repeat periprocedural evaluation again revealed no evidence of renal artery stenosis, sleep apnea, or an endocrine cause of hypertension. Laboratory testing was performed including routine blood work with hepatic and pancreatic enzymes, bilirubin, and renal function to enable structured safety surveillance.

### Ultrasound CHA-D

**Fig. 2:**
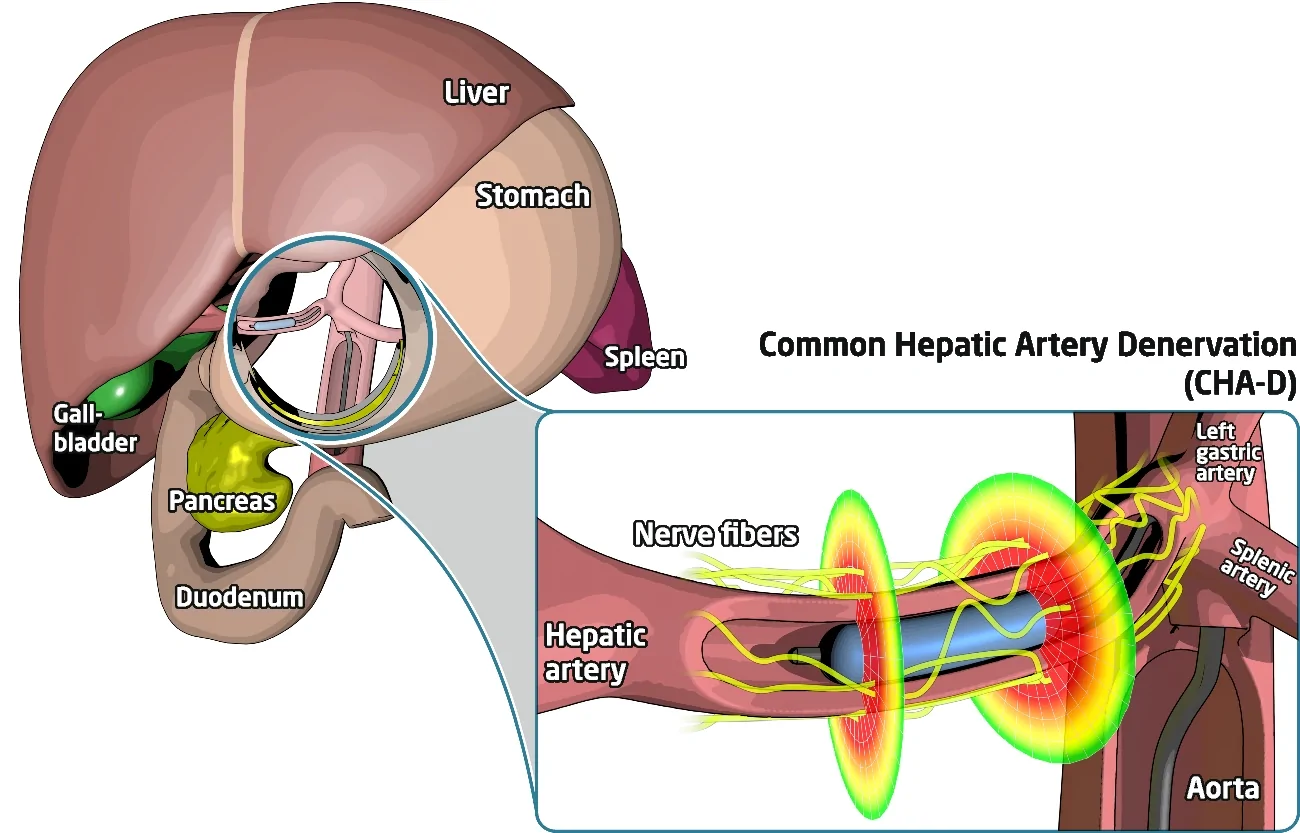
Schematic illustration of ultrasound CHA-D. Energy delivery targets periarterial sympathetic nerve fibers surrounding the vessel near the celiac trunk to achieve neuromodulation. CHA-D, common hepatic artery denervation

**Fig. 3:**
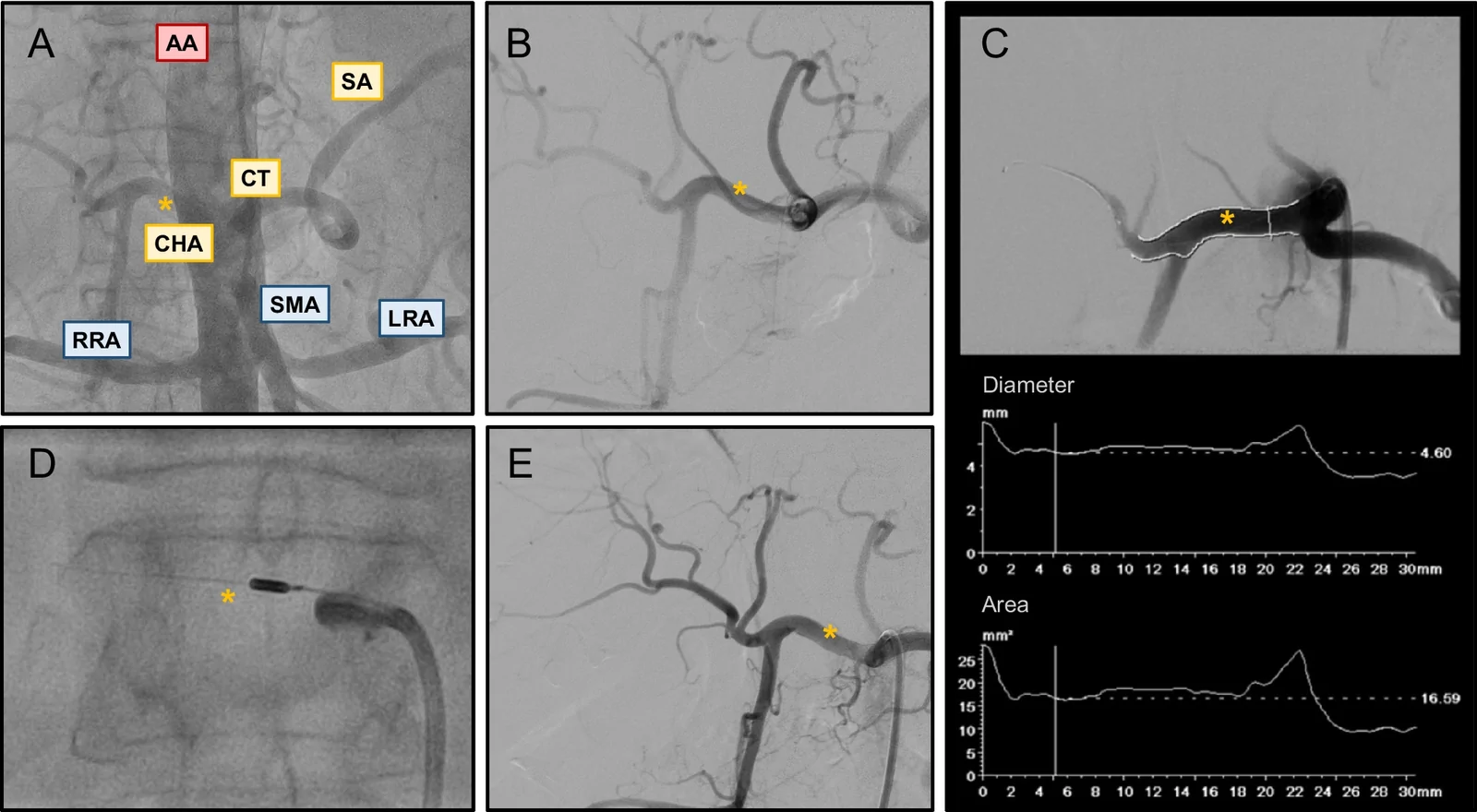
Angiographic guidance and catheter positioning during ultrasound CHA-D. Selective angiography of the celiac trunk and common hepatic artery (**A**, **B**), fluoroscopic assessment of the target segment and vessel dimensions (**C**), and positioning of the ultrasound denervation catheter in the target segment (**D**). Final angiographic control confirmed vessel patency and preserved branching pattern without apparent complications (**E**). The yellow asterisk indicates the common hepatic artery. CHA, common hepatic artery; CHA-D, common hepatic artery denervation; CT, celiac trunk; LRA, left renal artery; RRA, right renal artery; SA, splenic artery; SMA, superior mesenteric artery

The intervention was performed in an angiographic catheterization laboratory under continuous monitoring, including electrocardiography, oxygen saturation, and invasive BP measurements. Periprocedural anticoagulation consisted of 7500 IU unfractionated heparin with subsequent ACT-guided dosing to a target activated clotting time of 250 s. Analgesia was provided in a demand-adapted manner, particularly during energy delivery. Vascular access was obtained via ultrasound-guided puncture of the right common femoral artery using a 7 F, 10-cm Radifocus Introducer II sheath (Terumo, Tokyo, Japan) under local anesthesia. Selective catheterization of the celiac trunk for common hepatic artery access was performed using a 7 F LIMA guiding catheter (Boston Scientific, Marlborough, MA, USA). A 0.014-inch, 300-cm Iron Man guidewire (Abbott Vascular, Santa Clara, CA, USA) was advanced into the hepatic arterial tree to support catheter delivery and stable positioning of the Paradise ultrasound catheter (Recor Medical, Palo Alto, CA, USA). A total of 40 mL of iodinated contrast (Iomeron 300) was administered. Selective angiography of the celiac axis and the CHA was used to confirm vessel caliber, branching pattern, and the intended treatment segment. Bilateral renal artery angiography showed no renal artery stenosis, and angiography of the celiac axis demonstrated no relevant stenosis of the truncus coeliacus or its major branches. Ultrasound CHA-D was then performed using the Paradise ultrasound catheter (Recor Medical, Palo Alto, CA, USA). Figure 2 provides a schematic illustration of the CHA-D procedure. Energy delivery was applied in the distal CHA proximal to the gastroduodenal artery take-off and then sequentially more proximally toward the CHA origin from the celiac trunk (Fig. 3). In total, three ultrasound ablations were delivered. Device-specific safety parameters were continuously monitored via the system’s feedback functions, and energy delivery was executed in accordance with the device software and procedural protocol. After completion of energy delivery, final angiography was performed to document preserved flow and to exclude dissection, thrombus formation, vasospasm, or other angiographic abnormalities. No flow-limiting irregularities were observed.

### Post-procedural monitoring

Hemostasis was achieved using a femoral vascular closure device (FemoSeal), followed by a compression dressing. Because of aspirin intolerance, a loading dose of 300 mg clopidogrel was administered after the intervention, followed by 75 mg once daily for 4 weeks. Postinterventional surveillance included puncture-site assessment, hemodynamic monitoring, and evaluation for abdominal symptoms. Safety laboratory testing focused on hepatobiliary and pancreatic integrity and included hepatic enzymes (AST, ALT, γ-glutamyl transferase, and alkaline phosphatase), bilirubin, pancreatic enzymes (lipase and amylase), and renal function, with additional blood count or inflammatory markers obtained as clinically indicated.

### Blood pressure assessment and follow-up

For longitudinal assessment, ambulatory and office BP monitoring was performed at 1 and 6 months after the procedure. Echocardiographic follow-up was performed at 3 months. PDFF-MRI and the EQ-5D-5L and SF-36 questionnaires were repeated at 6 months to evaluate changes in hepatic steatosis and patient-reported health status. Routine blood sampling, including the predefined safety laboratory panel, was obtained at each follow-up visit.

### Endpoints and statistical analysis

The primary endpoint was procedural feasibility and safety, defined as successful completion of CHA-D without procedure-related serious adverse events, including clinically relevant vascular injury, acute hepatobiliary or pancreatic complications, or clinically meaningful laboratory derangements. Secondary exploratory endpoints included changes in 24-h ambulatory BP and office BP over follow-up, as well as longitudinal changes in laboratory surrogate markers reflecting hepatic, pancreatic, renal, and metabolic safety domains. Given the single-patient first-in-human design, results were reported descriptively, with continuous variables presented as absolute values and changes from baseline.

## Results

**Fig. 4:**
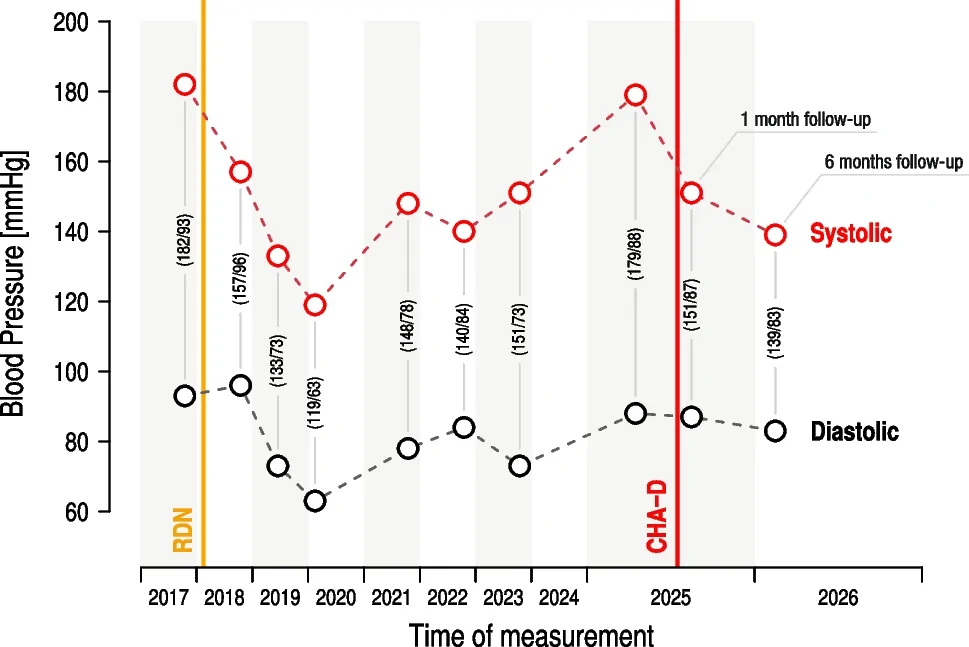
Longitudinal ambulatory blood pressure course before and after ultrasound CHA-D. Initial response to prior renal denervation, subsequent recurrence of hypertension, and the marked reduction in blood pressure following CHA-D are shown. CHA-D, common hepatic artery denervation

A 67-year-old woman was referred for evaluation of recurrent severe hypertension. Her medical history included type 2 diabetes mellitus, systemic lupus erythematosus in remission on stable low-dose prednisolone, and extensive medication intolerance/allergies, which had repeatedly limited escalation of antihypertensive therapy. For several years, felodipine 10 mg once daily had remained the only antihypertensive medication tolerated. Reported intolerances or allergies included among other ramipril, captopril, enalapril, candesartan, telmisartan, hydrochlorothiazide, chlorthalidone, and spironolactone. She had previously undergone catheter-based renal denervation in 2017, with a marked and durable ambulatory BP response over the subsequent years, reaching a nadir around 2020/2021. Thereafter, BP gradually increased again, culminating in a pronounced recurrence of uncontrolled hypertension in 2025 (Fig. 4). At presentation, the patient reported a substantial symptom burden consistent with heightened sympathetic activation, including impaired stress tolerance and headache. Given the inability to further escalate antihypertensive therapy because of extensive intolerance, and the clinically relevant BP deterioration after an initially favorable response to prior sympathetic modulation, CHA-D was performed after detailed discussion with and informed agreement of the patient.

### Procedural and clinical follow-up

The procedure was completed successfully without intraprocedural complications. Three ultrasound applications were delivered in the CHA as planned, and final angiography confirmed preserved vessel patency without dissection, thrombus formation, vasospasm, or any other flow-limiting abnormality. Serial laboratory testing showed no evidence of hepatobiliary, pancreatic, or renal injury after CHA-D (Table 1). Compared with pre-interventional values, liver enzymes remained stable at the early post-procedural assessment 1 day after CHA-D and during 1- and 6-month follow-up. Bilirubin, pancreatic enzymes, and renal function also showed no clinically relevant deterioration. At 6 months, renal function was slightly improved compared with baseline, with serum creatinine decreasing from 1.02 to 0.96 mg/dL and creatinine-based estimated glomerular filtration rate increasing from 56.7 to 60.7 mL/min/1.73 m^2^. In addition, the patient did not report abdominal complaints or other procedure-related symptoms at any time during follow-up. At 3-month echocardiographic follow-up, diastolic functional parameters improved, with a prolongation of deceleration time from 194 to 269 ms, a decrease in E velocity from 90 to 52 cm/s, a decrease in the E/A ratio from 0.9 to 0.6, and a reduction in E/E′ from 12.9 to 7.4, whereas other echocardiographic parameters remained stable.

**Table 1:** Serial safety laboratory parameters before and after ultrasound CHA-D

Parameter	Unit	Baseline	1 day after CHA-D	1-month follow-up	6-month follow-up
Aspartate aminotransferase	U/L	26	18	28	23
Alanine aminotransferase	U/L	24	19	28	18
Alkaline phosphatase	U/L	60	56	–	74
Total bilirubin	mg/dL	0.4	0.3	–	0.5
Gamma-glutamyl transferase	U/L	19	17	18	15
Pancreatic amylase	U/L	14	12	–	14
Lipase	U/L	25	22	–	25
Creatinine	mg/dL	1.02	0.75	1.12	0.96
Estimated glomerular filtration rate, creatinine-based	mL/min/1.73 m²	56.7	82.2	50.5	60.7
C-reactive protein	mg/L	3.5	2.4	0.7	< 0.6

*CHA-D*, common hepatic artery denervation

Patient-reported outcomes also improved over time. At 6-month follow-up, improvements were observed in three SF-36 domains, with social functioning increasing from 75 to 100, general health from 70 to 90, and mental health from 88 to 92. On the EQ-5D-5L, the patient only reported slight pain or discomfort at baseline while the remaining domains were reported as normal. By 6 months, no noticeable findings were reported across all domains. The EQ-VAS global health score also improved from 80 to 85.

### Blood pressure and metabolic effects

**Fig. 5:**
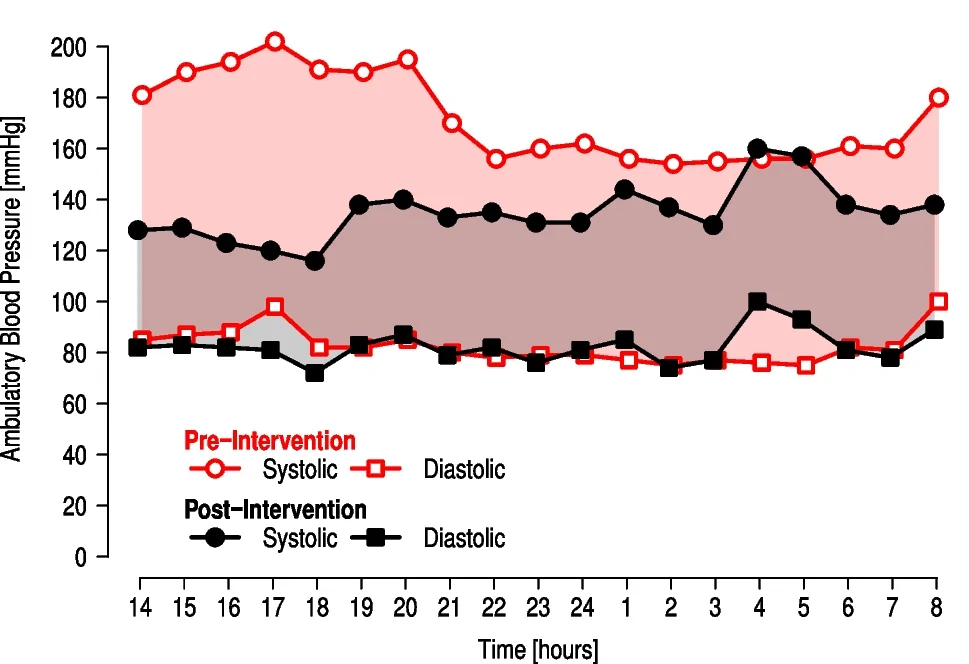
Ambulatory blood pressure profiles before and 6 months after ultrasound CHA-D. Systolic and diastolic blood pressure are shown across the day. CHA-D, common hepatic artery denervation

Ambulatory BP decreased substantially after the intervention (Figs. 4 and 5). Pre-procedural 24-h ambulatory BP measurement at presentation showed a mean BP of 179/88 mmHg. At 1 month, mean 24-h ambulatory BP declined to 151/87 mmHg, corresponding to a reduction of 28 mmHg in systolic BP. At 6 months, mean 24-h ambulatory BP was 139/83 mmHg, corresponding to a reduction of 40 mmHg in systolic and 5 mmHg in diastolic BP compared with baseline. Consistent with the ambulatory BP findings, office BP decreased from 176/116 mmHg at baseline to 149/87 mmHg at 1 month and to 125/85 mmHg at 6 months. These changes occurred without intensification of antihypertensive treatment, as the antihypertensive regimen remained unchanged throughout follow-up.

**Fig. 6:**
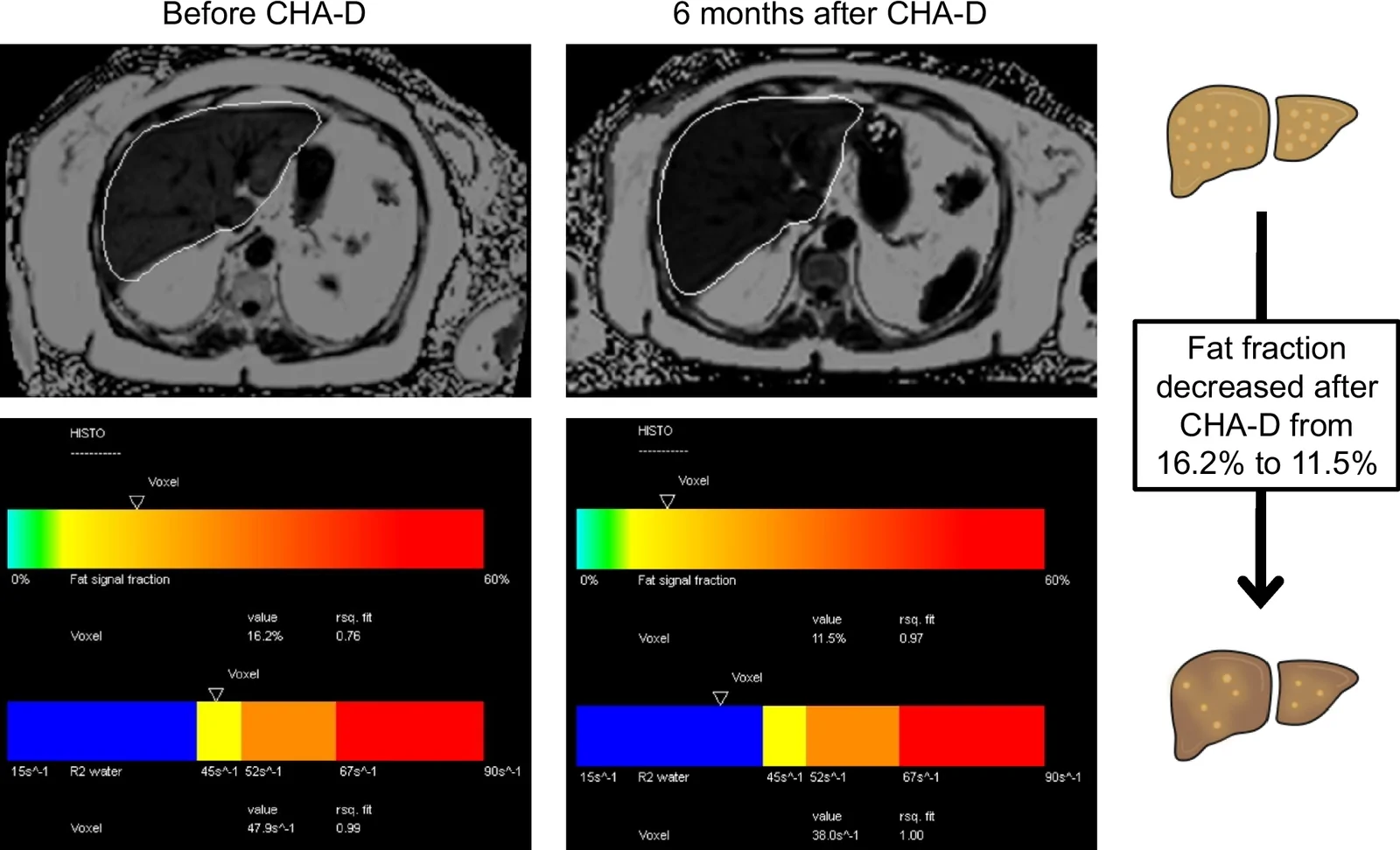
Reduction in hepatic fat fraction assessed by PDFF-MRI. Proton density fat fraction magnetic resonance imaging (PDFF-MRI) at baseline and 6-month follow-up demonstrating a reduction in hepatic fat content from 16.2 to 11.5%, corresponding to an absolute decrease of 4.7 percentage points

Beyond BP lowering, insulin requirement decreased, with prandial insulin aspart reduced from 3-4-3 to 2-1-2 IU and basal insulin detemir reduced from 24 to 16 IU. HbA1c also decreased from 6.2% at baseline to 5.9% at follow-up. In parallel, body weight decreased by 8 kg, while the patient reported no relevant change in dietary habits or physical activity. PDFF-MRI demonstrated a reduction in hepatic fat content from 16.2% at baseline to 11.5% at follow-up, corresponding to an absolute reduction of 4.7 percentage points and a relative reduction of approximately 29% (Fig. 6). The hepatic steatosis index, a noninvasive surrogate marker of hepatic steatosis, decreased from 44.4 at baseline to 39.3 at follow-up.

## Discussion

CHA-D was associated with marked reductions in ambulatory and office systolic BP of 40 and 51 mmHg, respectively, at 6 months despite unchanged antihypertensive therapy. Favorable cardiometabolic changes were also observed, including a reduction in MRI-derived hepatic fat fraction, lower insulin requirement, a decrease in HbA1c from 6.2 to 5.9%, and modest weight loss. This first-in-human study demonstrates that ultrasound CHA-D was technically feasible and not associated with procedural or safety concerns during short- to mid-term follow-up. Taken together, these findings provide the first-in-human evidence that ultrasound CHA-D may have both hemodynamic and metabolic relevance in carefully selected patients with uncontrolled hypertension and cardiometabolic comorbidity.

The BP response observed in this study is consistent with preliminary results that CHA-D or multi-organ denervation may contribute to BP lowering beyond renal denervation alone [19, 20]. In a multicenter, open-label, single-arm study of catheter-based endovascular denervation targeting the coeliac and hepatic arteries in patients with poorly controlled type 2 diabetes, office systolic BP decreased numerically by 4.8 mmHg at 6 months, while diastolic BP was reduced by 5.4 mmHg at 6 months [20]. The modest antihypertensive effect in that study is explained by the study population, which was enrolled primarily for diabetes rather than uncontrolled hypertension [20]. Preliminary findings from the NECTAR IV multi-organ denervation study further support this concept [19]. NECTAR IV enrolled patients with both uncontrolled type 2 diabetes and hypertension and evaluated combined CHA-D and RDN using the Neuviant MDN system [19]. This platform uses a dedicated catheter to deliver dehydrated ethanol into the periarterial tissue to achieve neurolysis [19], and thus differs mechanistically from the ultrasound-based system used in the present case. According to preliminary 6-month results, mean ambulatory BP decreased by 14.3/7.9 mmHg and mean office BP by 12.7/13.5 mmHg, while mean HbA1c was reduced by 1.0 percentage point [19]. These data remain preliminary and have not yet been confirmed in randomized sham-controlled trials, but they are encouraging and support the concept that multi-organ sympathomodulation may provide clinically meaningful antihypertensive and cardiometabolic effects. The mechanistic interpretation of the pronounced BP response requires caution, as the present report was not designed to directly assess autonomic mechanisms and lacked a control or sham procedure. Nevertheless, a CHA-D-related contribution is biologically plausible. CHA-D and RDN both aim to modulate periarterial sympathetic nerve activity, although they target different vascular territories. Human morphometric data demonstrate abundant sympathetic nerve fibers surrounding the common hepatic artery [14], supporting the anatomical plausibility of CHA-D as a neuromodulatory intervention. Mechanistic studies of RDN further suggest that interruption of afferent renal nerve signaling may attenuate central sympathetic outflow [23, 24]. Although an analogous afferent mechanism has not yet been demonstrated for CHA-D, this supports the broader concept that periarterial denervation may influence autonomic regulation beyond local organ effects. Experimental preclinical data further show that combined renal denervation and celiac ganglionectomy produce additive BP lowering [25]. Although celiac ganglionectomy is a more proximal intervention than CHA-D, these findings support the plausibility that modulation of an additional sympathetic territory may lower BP beyond prior RDN, potentially through reduced regional vascular resistance and increased vascular conductance [25, 26]. In the present patient, an additional individual factor may have heightened the relevance of sympathomodulation. Systemic lupus erythematosus contributes to autonomic dysregulation and persistent sympathoexcitatory drive [27, 28]. This may, at least in part, explain the pronounced initial response to prior renal denervation and the later recurrence of uncontrolled hypertension. In this context, further sympathomodulation in the form of CHA-D may help explain the renewed marked BP response to a sympathomodulatory intervention in this patient against the background of persistently elevated sympathetic tone.

Beyond BP lowering, the observed metabolic changes are of particular interest. Notably, HbA1c reduction was in a similar range to that reported with 10 mg empagliflozin [29], despite a concomitant reduction in insulin requirement. These findings are consistent with preclinical data showing that interruption of hepatic sympathetic signaling reduces steatosis and improves glucose tolerance, and with a broader body of evidence supporting a bidirectional interplay between sympathetic overactivity and metabolic syndrome, insulin resistance, and hepatic steatosis [15, 16, 30, 31]. Clinical experience with RDN also supports the concept that sympathomodulation may influence metabolism beyond BP alone, including favorable effects on glucose metabolism, insulin resistance, and hepatic steatosis [12, 13]. In addition, according to preliminary NECTAR IV 6-month data, mean HbA1c was reduced by 0.98% [19]. These findings suggest that CHA-D or multi-organ denervation may represent a cardiometabolic intervention with effects extending beyond pure hemodynamic modulation.

This first-in-human ultrasound CHA-D case should be interpreted within an evolving and heterogeneous clinical trial landscape. Several ongoing and planned studies are evaluating CHA-D and multi-organ sympathomodulation across hypertensive and metabolic populations, including combined RDN and CHA-D programs, CHA-D studies in type 2 diabetes, and ethanol- or radiofrequency-based neuromodulation approaches. Because these studies differ substantially in target population, technology, target anatomy, design, and endpoints, the present case should be viewed as early feasibility and safety data for ultrasound-based CHA-D, with exploratory hemodynamic and metabolic signals supporting further prospective evaluation of this emerging sympathomodulatory target. Key characteristics of the major ongoing studies are summarized in Table 2.

**Table 2:** Overview of ongoing clinical studies on hepatic and multi-organ denervation

Study	Target/approach	Design	Device	Outcomes
COMPLEMENT (NCT02278068)	Common hepatic artery denervation	Prospective, multicenter, non-randomized study in patients with poorly controlled T2D	Metabolic Neuromodulation System (MNS; RF energy)	Primary outcome: safety, mainly incidence of SADEs after RF hepatic denervation. Secondary outcomes: incidence of successful energy delivery, incidence of SADEs within 24 h, exploratory efficacy signals in glycemic control, including HbA1c and other metabolic measures in patients with poorly controlled T2D
DeLIVER (NCT04285554)	Common hepatic artery denervation	Prospective, single-arm, multicenter study in patients with T2D	Metavention Integrated Radio Frequency Denervation System (RF energy)	Primary outcome: safety, mainly incidence of SADEs after RF hepatic denervation. Secondary outcomes: changes in glycemic control, hepatic steatosis, and office BP
EDN-T2DM (NCT05673668)	Endovascular denervation (not further specified)	Prospective, single-center, single-arm, self-controlled study in patients with T2D	Netrod endovascular denervation system (RF energy)	Primary outcome: changes in HOMA-β/HOMA-IR at 6 months. Secondary outcomes: incidence of device or procedure-related major adverse events, safety, glycemic and metabolic endpoints, liver fat and liver function, clamp-based insulin sensitivity, biomarker profiling, microbiota/metabolome, and sympathetic activity
NECTAR III (NCT05363761)	Multi-organ denervation (hepatic + renal)	Prospective, multicenter, single-arm study in patients with T2D and hypertension	Neurotronic infusion catheter (ethanol-based neurolysis)	Primary outcomes: 30-day safety composite and device success. Secondary outcomes: glycemic control and BP response over follow-up
NECTAR IV (NCT05369065)	Multi-organ denervation (hepatic + renal)	Prospective, multicenter study with a randomized sham-controlled cohort plus a treatment cohort in patients with uncontrolled T2D and uncontrolled hypertension	Neurotronic infusion catheter (ethanol-based neurolysis)	Primary outcomes: 30-day safety composite, device success. Secondary outcomes: glycemic control, BP response, procedural success, and medication changes over follow-up
SPYRAL GEMINI pilot (NCT06907147)	Multi-organ denervation (hepatic + renal)	Prospective, multicenter, international pilot study with two parallel single-arm cohorts in uncontrolled hypertension (off antihypertensive medication vs on medication with high cardiovascular risk)	Gemini System with the Symplicity Spyral™ catheter (RF energy)	Primary outcome: change in office BP. Secondary outcomes: ABPM, home BP, BP target achievement, and comprehensive cardiovascular and organ-specific safety outcomes
MODUS (NCT04917393)	Multi-organ denervation (hepatic + renal)	Prospective, multicenter, single-arm, sham-controlled study in patients with hypertension and/or T2D	Metavention Integrated Radio Frequency Denervation System (RF energy)	Primary outcome: 30-day SADE rate. Secondary outcomes: office BP, ABPM, HbA1c, and fasting glucose over follow-up
DeLIVER NZ (ACTRN12619001524189)	Multi-organ denervation (hepatic + renal)	Prospective, single-arm, multicenter, non-randomized study in hypertensive adults with cardiometabolic risk features, including T2D/metabolic dysfunction	Metavention Integrated Radio Frequency Denervation System (RF energy)	Primary outcome: 90-day SADE rate. Secondary outcomes: glycemic, BP, anthropometric, adverse event, and hepatic laboratory outcomes

*ABPM*, ambulatory blood pressure monitoring; *BP*, blood pressure; *HbA1c*, hemoglobin A1c; *HOMA-β*, homeostasis model assessment of β-cell function; *HOMA-IR*, homeostasis model assessment of insulin resistance; *RF*, radiofrequency; *SADEs*, serious adverse device effects; *T2D*, type 2 diabetes

Safety is a major concern for any intervention involving the CHA because of its close anatomical proximity to hepatobiliary and pancreatic structures. In the present case, there were no angiographic complications, no post-procedural abdominal symptoms, and no biochemical signs of hepatobiliary, pancreatic, or renal injury during serial follow-up. Patient-reported outcomes also improved over follow-up, indicating an improvement in health-related quality of life.

### Study limitations

As a single compassionate-use case, this report cannot establish efficacy, causality, or generalizability. However, it offers the first comprehensive human description of the BP and cardiometabolic effects associated with ultrasound CHA-D. The patient’s complex clinical background, including prior RDN, type 2 diabetes, systemic lupus erythematosus, and extensive medication intolerance, could limit extrapolation to specific populations. Although antihypertensive therapy remained unchanged and metabolic changes occurred consistently across several domains, including reduced hepatic fat fraction, lower HbA1c, reduced insulin requirement, lower hepatic steatosis index, and body weight reduction, the contribution of unmeasured lifestyle-, treatment-, or disease-related factors to the observed hemodynamic and metabolic changes cannot be fully excluded. Prospective randomized controlled studies are required to further investigate the independent hemodynamic and metabolic effects of CHA-D.

## Conclusion

This first-in-human study shows that ultrasound CHA-D is technically feasible and was performed with a favorable short- to mid-term safety profile in a treatment-limited patient with recurrent uncontrolled hypertension and cardiometabolic comorbidity. The parallel improvements in ambulatory and office BP, hepatic fat fraction, HbA1c, body weight, and insulin requirement support the concept that CHA-D may represent a clinically meaningful cardiometabolic intervention rather than an approach confined to BP reduction alone. These findings identify CHA-D as a promising adjunctive sympathomodulatory strategy and warrant prospective clinical evaluation in patients with hypertension and cardiometabolic disease.

## Data Availability

All data related to this study are available from the corresponding author on reasonable request.

## Abbreviations

BP: Blood pressure
CHA: Common hepatic artery
CHA-D: Common hepatic artery denervation
HbA1c: Glycated hemoglobin A1c
PDFF-MRI: Proton density fat fraction magnetic resonance imaging
RDN: Renal denervation
